# SpaK/SpaR Two-component System Characterized by a Structure-driven Domain-fusion Method and *in Vitro* Phosphorylation Studies

**DOI:** 10.1371/journal.pcbi.1000401

**Published:** 2009-06-05

**Authors:** Anu Chakicherla, Carol L. Ecale Zhou, Martha Ligon Dang, Virginia Rodriguez, J. Norman Hansen, Adam Zemla

**Affiliations:** 1 Computing Applications and Research Department, Lawrence Livermore National Laboratory, Livermore, California, United States of America; 2 Sacred Hearts Academy, Honolulu, Hawaii, United States of America; 3 Genome Technology Branch, National Human Genome Research Institute, National Institutes of Health, Bethesda, Maryland, United States of America; 4 Department of Chemistry and Biochemistry, University of Maryland, College Park, Maryland, United States of America; National Institutes of Health, United States of America

## Abstract

Here we introduce a quantitative structure-driven computational domain-fusion method, which we used to predict the structures of proteins believed to be involved in regulation of the subtilin pathway in *Bacillus subtilis*, and used to predict a protein-protein complex formed by interaction between the proteins. Homology modeling of SpaK and SpaR yielded preliminary structural models based on a best template for SpaK comprising a dimer of a histidine kinase, and for SpaR a response regulator protein. Our LGA code was used to identify multi-domain proteins with structure homology to both modeled structures, yielding a set of domain-fusion templates then used to model a hypothetical SpaK/SpaR complex. The models were used to identify putative functional residues and residues at the protein-protein interface, and bioinformatics was used to compare functionally and structurally relevant residues in corresponding positions among proteins with structural homology to the templates. Models of the complex were evaluated in light of known properties of the functional residues within two-component systems involving His-Asp phosphorelays. Based on this analysis, a phosphotransferase complexed with a beryllofluoride was selected as the optimal template for modeling a SpaK/SpaR complex conformation. *In vitro* phosphorylation studies performed using wild type and site-directed SpaK mutant proteins validated the predictions derived from application of the structure-driven domain-fusion method: SpaK was phosphorylated in the presence of ^32^P-ATP and the phosphate moiety was subsequently transferred to SpaR, supporting the hypothesis that SpaK and SpaR function as sensor and response regulator, respectively, in a two-component signal transduction system, and furthermore suggesting that the structure-driven domain-fusion approach correctly predicted a physical interaction between SpaK and SpaR. Our domain-fusion algorithm leverages quantitative structure information and provides a tool for generation of hypotheses regarding protein function, which can then be tested using empirical methods.

## Author Summary

Because proteins so frequently function in coordination with other proteins, identification and characterization of the interactions among proteins are essential for understanding how proteins work. Computational methods for identification of protein-protein interactions have been limited by the degree to which proteins are similar in sequence. However, methods that leverage structure information can overcome this limitation of sequence-based methods; the three-dimensional information provided by structure enables identification of related proteins even when their sequences are dissimilar. In this work we present a quantitative method for identification of protein interacting partners, and we demonstrate its use in modeling the structure of a hypothetical complex between two proteins that function in a bacterial signaling system. This quantitative approach comprises a tool for generation of hypotheses regarding protein function, which can then be tested using empirical methods, and provides a basis for high-throughput prediction of protein-protein interactions, which could be applied on a whole-genome scale.

## Introduction

Because proteins so frequently function in coordination with other proteins, identification and characterization of protein-protein complexes are essential aspects of protein sequence annotation and function determination [1]. A variety of empirical [2]–[4] and computational [5]–[14] methods for identifying putative protein-protein interactions have been reported. Of particular note is the Rosetta Stone approach for identifying interacting partners based on the theory of gene fusion, whereby protein domains that are encoded separately in one species may be homologous to domains that are “fused” in the same open reading frame in another species [15]–[17]. Whereas sequence-based domain fusion methods can be highly successful in identifying putative functional relationships among proteins, the reliance on sequence homology limits detection to protein sequences with adequate levels of sequence identity. Another approach to identifying putative protein-protein interactions is described by Lu and coworkers [18], whereby sequence-based searches against the PDB database were performed in order to identify multi-domain structures having at least one domain with good sequence identity to each putative interacting protein. However, the sensitivity of this search method is also dependent on the levels of sequence identity between the proteins of interest and the sequences of the domains within the identified PDB domain-fusion template. Kundrotas and Alexov [6] explored the use of structure-based comparisons in the identification of multi-domain templates for homology modeling of complex structures. In this work, it was determined that a structure-based protocol performed considerably better than did a sequence-based protocol in recovering known protein-protein interacting partners (86% recovery as opposed to 19%) in searches against a database of known complexes, indicating that the structure-based method was more sensitive in detecting remote homologs.

We describe the application of a quantitative structure-based comparison method to the identification of putative protein-protein interactions, and show that this approach increases sensitivity in detecting putative interactions at low (<20%) levels of sequence identity, based on the general principle that structure homology is more highly conserved in evolution than is sequence homology [19]. Our approach, therefore, involves the generation of a structure model, based on adequate (typically >30%) sequence identity to a PDB domain, followed by structure-based homology searches against PDB to identify multi-domain structures with adequate structure identity [20] to the model of each putative interacting protein. Thus, we propose that our structure-driven domain-fusion method can be used to identify domain-fusion templates for modeling protein-protein interaction complexes, and that such searches may prove to be more sensitive than sequence-based searches alone.

To explore this approach, we selected as the subject of our study a protein-protein interaction that is representative of a common class of biological control systems, known as the two-component signal transduction system [21]–[24]: the interaction of SpaK and SpaR from *Bacillus subtilis*, which regulate the biosynthesis of subtilin, an antimicrobial peptide lantiobiotic that inhibits growth of a broad range of pathogenic Gram positive bacteria [25]–[27]. In this study we introduce a structural bioinformatics methodology for identification of putative protein-protein complexes, and we apply it to characterize the interactions between SpaK and SpaR. We generate structure homology models of SpaK and SpaR, and then use these models to identify multi-domain protein structures that have good structure homology to the models. Using one of the so-identified domain-fusion templates, we generate a model representing a hypothetical physical interaction between SpaK and SpaR, which enables further analyses of residues involved in the protein-protein interaction. In this way we extend the well-known sequence-based domain-fusion method by leveraging structural data, and use it to generate hypotheses regarding the interactions between the two proteins. We further report the results of biochemical studies on wild type and mutant proteins that characterize the interactions between SpaK and SpaR, and we assess the resulting structural model of a putative SpaK/SpaR complex arising from our structure-driven domain-fusion approach. Furthermore, our biochemical analyses confirm that SpaK autophosphorylates and subsequently transfers a phosphoryl group to SpaR.

## Materials and Methods

### Homology modeling of SpaK and SpaR proteins

SpaK (gi: 6226707, Uniprot P33113) and SpaR (gi: 417799, Uniprot P33112) protein sequences were input to the AS2TS protein structure modeling system ([28]; http://as2ts.llnl.gov/), which generated initial homology models based on structures taken from the Protein Databank (PDB) (version released December 11, 2007). Structural templates having global sequence homology to each of SpaK and SpaR were further studied by examining domain-level homology.

**Figure 1:**
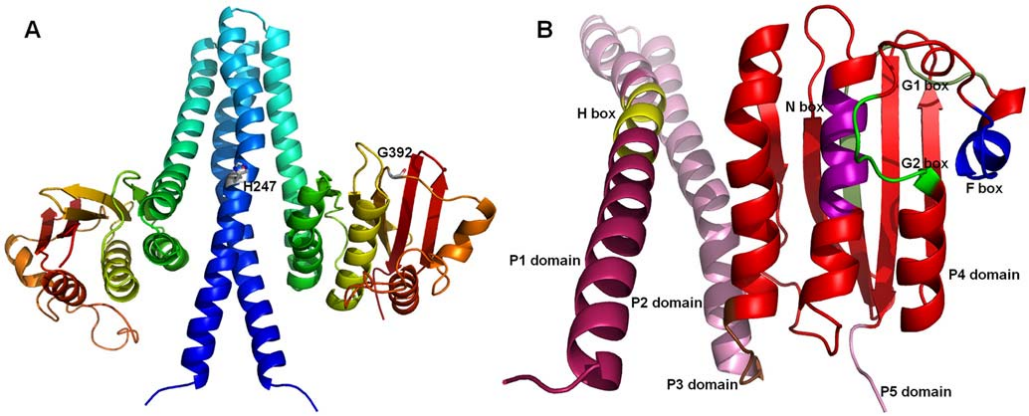
Homology model of SpaK based on PDB entries 2c2a and 2ftk. Modeled region: 219–459. The 218-residue long N-terminal membrane spanning region (residues 1–218) was not modeled. A: Model of the oligomeric state: homodimer. Coloring scheme reflects in each modeled monomer a consecutive ordering of amino acids in the N-to-C-terminal direction, whereby N-most residues are colored blue and C-most residues are red. Blue-cyan (residues 219–300): central four-helix bundle formed by interaction of 2 helixes from each monomer; Green-red (residues 301–459): C-terminal ATPase-c domain. The labels H247 and G392 show the location of two residues that were changed using site-directed mutagenesis to construct mutants for the phosphorylation studies (see Materials and Methods). B: Homology model of SpaK with marked domains: P1 (dark pink; 219–254), P2 (pink helix; 255–305), P3 (brown; 306–310), P4 (red; 311–455), and P5 (pink strand; 456–459) that are considered as 5 separate functional units. Characteristic sequence motifs (“boxes”) are colored as follows: H (yellow), N (plum), G1 (pale green), F (blue), and G2 (green). Highlighted motifs correspond to those in Fig. 1 from [41] (see Table 3).

As no suitable template for the N-terminal domain (218 residues) of SpaK was identified, this domain was not modeled. Based on match length (227 residues), e-value (4e-57), and sequence identity (28%), PDB entry 2c2a_A, a sensor histidine kinase from *Thermotoga maritima*, was identified as the primary template for modeling SpaK (Fig. 1). Additional templates identified by AS2TS are shown in Supplemental Results Table S1. Two domains of SpaK (SpaK_d1: residues 219–300 and SpaK_d2: 301–459) were modeled separately, pending determination of relative conformation to be provided by structure-driven domain-fusion analysis (see Results). Although identification of a structure template with acceptable global sequence homology enables initial model construction, there often remain sub-sequences in the protein of interest that do not correspond to any portion of the template due to insertions or deletions relative to that template. For this reason, and in order to construct as complete a model as possible to confirm the fitness of the modeled complex, the Local-Global Alignment (LGA) modeler gap-filling procedure (in-house software) was used to construct necessary loops, gaps or insertions by “grafting” in suitable regions from related structures in PDB.

**Figure 2:**
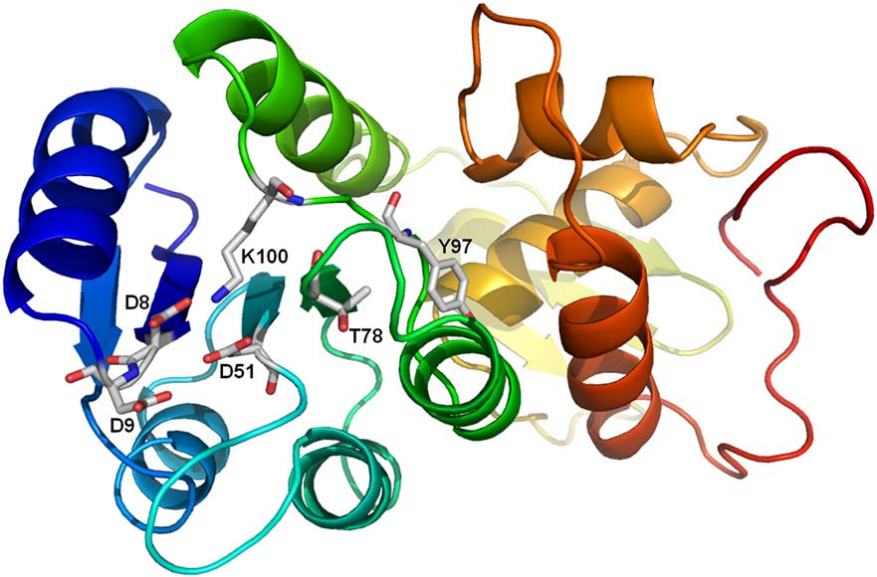
Homology model of the SpaR N-terminal (residues 1–117) and C-terminal (residues 118–220) domains. Modeling of the N-terminal domain was based on PDB template 1mvo_A, and the C-terminal domain was based on PDB template 2gwr_A. The conformation between domains was modeled based on 2gwr (response regulator protein MTRA from *Mycobacterium tuberculosis*). Coloring scheme reflects consecutive ordering of amino acids from the N-terminal region (blue) to the C-terminal region (red). Residues in SpaR that correspond to the functional residues in response regulator 2ftk (Spo0F; see Table 2B) are displayed as sticks.

Similarly, SpaR was modeled as two separate domains, comprising residues SpaR_d1: 1–117 and SpaR_d2: 118–220. The N-terminal domain was initially modeled based on the structural template 1mvo_A (crystal structure of the PhoP receiver domain from *Bacillus subtilis*), which showed the highest level of sequence identity (46%) to that domain (see Supplemental Results Table S2). In order to complete the model, the LGA gap-filling procedure was used to construct regions of missing coordinates. PDB entry 2gwr_A, a response regulator protein from *Mycobacterium tuberculosis*, was identified as the primary template for homology modeling of the C-terminal domain of SpaR (match length 216, e-value 9e-58, sequence identity 30%). This template was also used for the construction of the domain orientation (Fig. 2). Further refinement of the constructed SpaK and SpaR models was performed based on the structure comparison of modeled domains with other PDB templates that were structurally identified by a PDB-search procedure using LGA and the PDB release of July 8, 2008. In all created models the positioning of the sidechains for residues that were identical in the template were copied to the models, and the coordinates for missing side chain atoms were predicted using SCWRL [29].

### Structure-driven domain-fusion template identification

The LGA software ([20], http://as2ts.llnl.gov/lga/) was used to perform structure homology searches against the PDB database to identify all entries with detected (LGA_S> = 35%) structural similarity to any of the four modeled domains (see above) within the homology models of SpaK and SpaR. We selected an LGS_S cutoff value of 35% based on our observation that the number and quality of hits increased rapidly at LGA_S< = 33% (data not shown) and based on previous work [30] that determined the minimal structure homology needed to assure quality of structure alignment. Those entries with homology to both respective domains of SpaK and SpaR were selected as putative domain-fusion templates for modeling a SpaK/SpaR complex (Table 1). Reported in Table 1 are the sequence identities between SpaK or SpaR compared to each corresponding domain-fusion template, whereby residue-residue correspondences were extracted from the structure alignments between the models and the domain-fusion templates. We do not report the PSI-BLAST calculated sequence identities, as these are highly inaccurate and meaningless when calculated from sequence alignments at low levels of sequence identity (i.e., below 10%).

**Table 1:** Candidate domain-fusion templates for structure modeling of a SpaK/SpaR complex.

Template - SpaK/R^1^^,^ 2	N1^3^	N2^4^	N^5^	RMSD^6^	Seq_ID^7^	LGA_S^8^
1f51_A - SpaK_d2	181	159	104	2.56	6.73	42.32
1f51_E - SpaR_d1	119	117	116	1.41	25	93.11
2ftk_A - SpaK_d2	181	159	106	2.58	6.6	42.86
2ftk_E - SpaR_d1	119	117	116	1.11	24.14	95.71
1th8_A - SpaK_d2	132	159	95	2.34	17.89	42.99
1th8_B - SpaR_d1	115	117	76	2.71	7.89	39.3
1thn_A - SpaK_d2	136	159	99	2.23	17.17	45.15
1thn_B - SpaR_d1	114	117	75	2.75	6.67	38.68
1tid_A - SpaK_d2	136	159	98	2.23	17.35	44.47
1tid_B - SpaR_d1	119	117	76	2.88	6.58	38.52
1til_A - SpaK_d2	141	159	101	2.19	16.83	45.47
1til_B - SpaR_d1	117	117	71	2.96	4.23	37.11

1 The domains from the structure models of SpaK and SpaR were compared with all structures from PDB. Listed are those domain-fusion templates for which at least one domain from each of SpaK and SpaR had structure similarity LGA_S> = 35%.

2 The residue ranges in modeled SpaK domains are: SpaK_d1: 219–300 and SpaK_d2: 301–459, and the residue ranges in modeled SpaR domains are: SpaR_d1: 1–117 and SpaR_d2: 118–220.

3 N1 denotes a number of residues in the structural domain-fusion template.

4 N2 denotes the number of residues in the corresponding domain from SpaK or SpaR.

5 N denotes the number of superimposed C-alpha atoms that fit under a distance of 5.0 Angstroms.

6 RMSD is the root mean square deviation of N corresponding C-alpha atom pairs from the calculated structural alignment.

7 Seq_ID denotes the sequence identity in % between the domain-fusion template and the corresponding SpaK or SpaR domain calculated from the structural alignment.

8 LGA_S is a measure of the level of structure similarity [20] identified between the domain-fusion template and the corresponding domain from SpaK or SpaR.

Domains from the structural models of SpaK and SpaR were compared with all structures from PDB. Listed are the domain-fusion templates that for at least one domain from the SpaK or SpaR model had a level of structure similarity LGA_S above 37%. LGA_S scores are reported for alignments between each modeled domain of SpaK or SpaR and a domain-fusion template domain. The residue ranges in modeled SpaK domains were: SpaK_d1: 219–300 and SpaK_d2: 301–459, and the residue ranges in modeled SpaR domains were: SpaR_d1: 1–117 and SpaR_d2: 118–220.

### Cloning and expression of histidine-tagged proteins SpaK and SpaR

The *spa*K and *spa*R genes were isolated from *Bacillus subtilus* strain LH45, a subtilin-producing derivative of strain 168 [31]. Synthetic oligonucleotide primers were used to amplify *spa*R using methods described previously [32],[33]. Briefly, the commercial vector pQE31 (obtained from Qiagen, Valencia, CA), was digested with EcoRI and HindIII, and a fragment containing a truncated *spa*K gene encoding the C-terminal half of SpaK was cloned into the multipurpose cloning site of the QE31 vector to construct the pQE31-*spa*K expression vector (Supplemental Fig. S1A). (Note that we succeeded in expressing only the C-terminal residues of SpaK, as the full-length gene did not yield an expression product.) The pQE31-*spa*R vector was similarly constructed (details are shown in Supplemental Fig. S1B. Vectors (MLD[pQE31-*spa*R] and MLD[pQE31-*spa*K]) were transformed into JM109. For expression of the histidine-tagged proteins, the expression plasmids MLD[pQE31-*spa*K] and MLD[pQE31-*spa*R] were transformed into M15[pREP4] competent cells (Qiagen), and expressed according to the manufacturer's protocol. Expressed His-tagged proteins were purified using a Ni-NTA resin from Novagen to form slurries that were used to pack a 1.6 cm column, and eluted proteins were dialyzed against a storage buffer and stored in 50-ul aliquots at 80°C. A working stock was stored for several weeks at 20°C. Protein concentrations were determined by Bio-Rad protein assay using the manufacturer's protocol.

### Construction of mutant SpaK proteins

Mutant SpaK proteins were prepared by Ana-Gen Technologies (Palo Alto, CA) using the Stratagene QuikChange Mutagenesis Kit. Synthetic forward and corresponding reverse complement oligonucleotide primers were prepared for each of two mutations introduced into SpaK (altered nucleotides are indicated in bold type): at position H247 the histidine was changed to glutamine using forward primer 5′-GTGCTTTGGCACA**A**GAGATCAAGATTCCG-3′ and reverse primer: 5′-CGGAATCTTGATCTC**T**TGTGCCAAAGCAC-3′, and at position G392 the glycine was changed to alanine using forward primer 5′-GTAAAAGACACGG**C**AAATGGATTTTCGG-3′ and reverse primer 5′-CCGAAAATCCATTT**G**CCGTGTCTTTTAC-3′.

### In vitro phosphorylation and de-phosphorylation assays

Phosphorylation reactions were performed with each histidine-tagged SpaK wild type and mutant protein in the absence and presence of histidine-tagged SpaR. Upon addition of 32P-labeled ATP, reaction mixtures were incubated for 20 minutes at room temperature, after which the reactions were stopped by addition of 5× phosphorylation sample buffer, then electrophoresed on a 12.5% SDS polyacrylamide gel. The gel was stained with Coomassie blue, dried, and autoradiographed using Kodak X-OMAT AR film.

Phosphorimage analysis was performed to quantify incorporation and turnover of phosphate in assays involving phosphorylation of 6xHis-SpaK. Four samples of protein were incubated in the presence of 32P-labeled ATP, of which three were followed by cold chase treatment with unlabeled 4 mM, 10 mM, or 50 mM ATP, using reaction conditions described previously [34]. Samples were run on a 12.5% SDS-PAGE gel and subjected to autoradiography (not shown) and phosphorimaging. Image intensities of the radiolabeled-phosphorylated SpaK gel bands were analyzed using the Molecular Dynamics Phosphorimager 400.

Thin-layer chromatography was performed using Polygram Cell 300 PEI cellulose plates as described previously [35]. 6xHis-SpaK and 6xHis-SpaR were incubated individually (SpaK) or in combination with 32P-labeled ATP in the absence or presence of EDTA. One ul aliquots from each reaction were spotted onto TLC plates, and chromatography was carried out in 0.75 M KH_2_PO_4_, pH 3.75, after which the plate was dried and autoradiographed.

## Results

### Structure-driven domain-fusion analysis and protein-protein complex modeling

**Figure 3:**
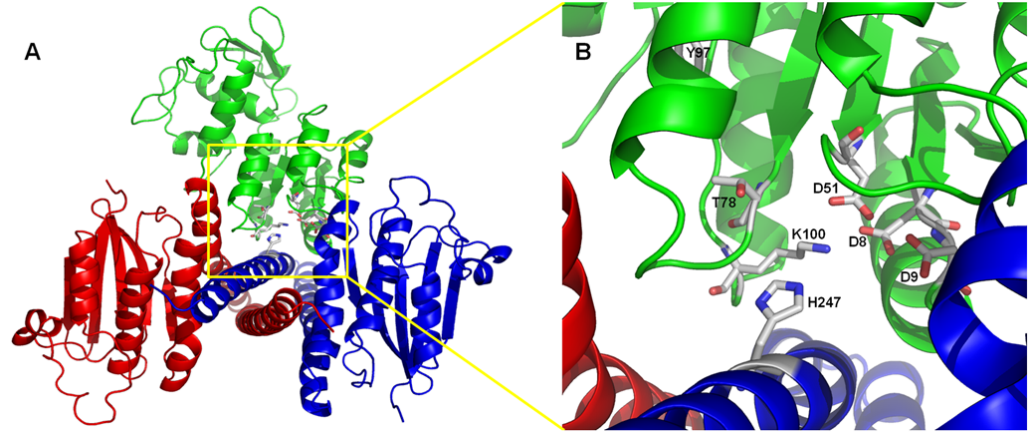
Homology model of a SpaK-SpaR complex. A: Model is based on the A and E chains of SPO0B, a phosphotransferase, complexed with SPO0F, a beryllofluoride (PDB template 2ftk). Blue, red: monomers of SpaK; Green: SpaR. B: Close up view of interacting residues (SpaK: H247; SpaR: D8, D9, D51; shown as stick) believed to mediate transfer of phosphate group from SpaK to SpaR.

The AS2TS protein structure modeling system [28] yielded over 30 and over 140 PDB structures suitable as templates for modeling each of SpaK and SpaR, respectively, from which were selected sets of the closest templates with sequence identities ranging from 13% to 28% for SpaK and 24% to 46% for SpaR (see Supplemental Data Tables S1, S2). LGA-mediated structure homology searches against the PDB database using constructed structural models of domains from SpaK (SpaK_d1, SpaK_d2) and SpaR (SpaR_d1, SpaR_d2) yielded 6 domain-fusion templates with structural homology (i.e., similarity based on structure alignment; [20]) ranging from LGA_S = 37% to 95%, and root mean square deviation (RMSD) calculated on superimposed C-alpha atoms ranging from 1.11 to 2.96 (Table 1). Identification of domain-fusion templates suggested that SpaK and SpaR interact forming an interface between domain 2 of SpaK and domain 1 of SpaR. Sequence identities of SpaK and SpaR to corresponding template sequences ranged from 4% to 25%, but in no instance was sequence identity greater than 7% simultaneously to both SpaK_d2 and SpaR_d1. Structural comparison of all identified domain fusion template structures showed that they clustered into two distinct conformations, yielding the following groups: (1) 1f51_AE and 2ftk_AE (Spo0F/Spo0B from *B. subtilus*), and (2) 1th8_AB, 1thn_AB, 1tid_AB and 1til_AB (SpoIIAB/SpoIIAA from *B. stearothermophilus*). PDB entry 2ftk was determined to be the optimal domain-fusion template for modeling a SpaK/SpaR complex based on the highest structure similarity to the corresponding two modeled domains: SpaK_d2 and SpaR_d1, and based on the expected intermolecular distance between the putative functional residues H247 of SpaK and D51 of SpaR that were predicted as active site residues (His and Asp) critical for exchanging a phosphoryl group [36]. In order to form a covalent bond with the phosphoryl group, the distances between atoms N of His and O of Asp were expected to be in the range of about 5 Angstroms. The models created based on templates 1f51 and 2ftk satisfied this requirement. 2ftk was also used to complete the homology model of SpaK (Fig. 1) by providing relative positioning of the central (SpaK_d1) and C-terminal (SpaK_d2) domains. The SpaK/SpaR complex was modeled as a trimer, comprising a SpaK homo-dimer and a SpaR monomer, based on the domain conformation between chains A and E from 2ftk (Fig. 3). The constructed model of a SpaK/SpaR complex agreed with structural analysis of the Spo0F and Spo0B interaction reported by Varughese and coworkers [37], who showed that the geometry of Spo0F binding to Spo0B favors an associative mechanism for phosphoryl transfer. In order to visualize the autophosphorylation of the histidine kinase, and the subsequent phosphoryl transfer to Spo0F, they generated *in silico* models representing these reaction steps, proposing Spo0B as a model for the autokinase domain of KinA (histidine kinase, consisting of an N-terminal sensor domain and a C-terminal autokinase domain). The level of sequence identity between KinA and SpaK is about 27%, and the KinA sensor domain comprises three PAS (Per-Arnt-Sim) domains that correspond to the N-terminal part of SpaK (1–218; not modeled). The autokinase domain corresponds to the modeled C-terminal part (219–459) of SpaK, and consists of a phosphotransferase subdomain and an ATP binding subdomain. In modeling SpaK we followed Varuguese and coauthors' suggestion that the four-helix bundle of Spo0B is formed through the dimerization of two helical hairpins from two monomers, and that it is a prototype for the phosphotransferase domains of histidine kinases (see Fig. 1A). This concept is supported by the high degree of structure similarity between the C-terminal domain of Spo0B and the ATP binding domains of histidine kinases, as well as by a report [38] of the crystal structure of the entire cytoplasmic portion of a histidine kinase (a PDB structure, 2c2a), which we used as a primary template for modeling individual domains of SpaK.

### Informatics analysis of functional residues and sequence motifs in a hypothetical SpaK/SpaR complex

Inspection of the constructed SpaK/SpaR complex (Fig. 3A) allowed us to identify specific residues putatively involved in the interaction between SpaK and SpaR or believed to mediate transfer of phosphate from SpaK to SpaR (Fig. 3B). Specifically, we identified the histidine residue at position H247 in SpaK that corresponds to the histidine H30 that is phosphorylated in Spo0B (PDB entry 2ftk_A) (Table 2A), and we identified 3 aspartate residues in close proximity in SpaR (D8, D9, and D51), which we presumed to be involved in transfer of a phosphoryl group bound to the H247 residue of SpaK, if SpaK and SpaR truly mediate a phosphorelay as postulated. These residues corresponded to their equivalents (D10, D11, and D54) in Spo0F (PDB entry 2ftk_E) (Table 2B). Three additional functional residues were identified, which corresponded to functional residues that are highly conserved among response regulator proteins [37]: T78, Y97, and K100 in SpaR, corresponding to T82, H101, and K104, respectively, of Spo0F (Table 2B). Under global superposition, the distances between corresponding functional residues were below 0.8 Angstroms and the local RMSD(3) (root mean square deviation along the main-chain atoms (N,CA,C,O) averaged over three residues: current and immediate neighbors along peptide chain (local superposition); [20]) values were below 0.5 Angstrom, indicating significant structure similarity in corresponding regions. The sites of phosphorylation, D51 of SpaR and H247 of SpaK, which correspond to D54 of Spo0F and H30 of Spo0B, are shown in Figure 3.

**Table 2:** Residue-residue correspondences between functional motifs in domain-fusion template 2ftk and SpaK (A) or SpaR (B) homology models.

A					
2ftk_A		SpaK			
Res^1^	ResName^2^	Res	ResName	Distance^3^	RMSD(3)^4^
R	29_A	A	246_A	0.508	0.14
**H**	30_A	**H**	247_A	0.565	0.236
D	31_A	E	248_A	0.644	0.203

B					
2ftk_E		SpaR			
Res	ResName	Res	ResName	Distance	RMSD(3)
V	1209_E	V	7_E	0.366	0.14
**D**	1210_E	**D**	8_E	0.433	0.191
**D**	1211_E	**D**	9_E	0.684	0.223
Q	1212_E	E	10_E	0.797	0.244
L	1253_E	L	50_E	0.277	0.221
**X** 5	1254_E	**D**	51_E	0.561	0.205
M	1255_E	V	52_E	0.731	0.178
M	1281_E	L	77_E	0.602	0.285
**T**	1282_E	**T**	78_E	0.78	0.398
A	1283_E	A	79_E	0.927	0.781
T	1300_E	D	96_E	1.276	0.417
**H**	1301_E	**Y**	97_E	0.737	0.367
F	1302_E	I	98_E	0.799	0.199
A	1303_E	T	99_E	0.832	0.27
**K**	1304_E	**K**	100_E	0.474	0.413
P	1305_E	P	101_E	0.366	0.509

1 Residue.

2 Residue name in PDB or model file.

3 Distance between C-alpha carbons (under global superposition).

4 RMSD(3): Root mean square deviation along the mainchain atoms (N,CA,C,O) averaged over three residues: current and immediate neighbors along peptide chain (local superposition).

5 X – aspartic acid (ASP) modified to aspartate beryllium trifluoride (BFD).

2ftk_A corresponds to Spo0B, and 2ftk_E corresponds to Spo0F. Letters in bold represent corresponding functional residues. Neighboring residues within 1 position of functional residues are included in order to provide a sequence-structure context in which highlighted residues were located. A) Residue-residue correspondences between histidine phosphorylation site and neighboring residues of 2ftk chain A and those of SpaK. B) Residue-residue correspondences between regions containing 6 functional residues of 2ftk chain E and SpaR.

In most histidine kinases the extracellular sensing domains are variable in sequence, reflecting the wide range of environmental signals to which they respond. Conversely, the cytoplasmic portions typically have a conserved catalytic core comprising a set of characteristic sequence motifs known as the H, N, G1, F and G2 boxes [39],[40] and can be dissected into several distinct functional units [41],[42]. Corresponding functional units P1 through P5 were evident upon examination of residues 219 through 459 of our modeled SpaK protein (Fig. 1B), which were determined to comprise an N-terminal dimerization and histidine phosphotransfer domain (DHp; SpaK_d1) and a C-terminal catalytic and ATP-binding domain (CA; SpaK_d2). P1 had a conserved histidine residue (H247) belonging to the autophosphorylation site known as the “H box”. Autophosphorylation was presumed to occur from ATP in the active site of P4 (the kinase domain) to H247 of P1, followed by transphosphorylation from H247 to an aspartate residue (D51) of SpaR. P2 functional units have a specific domain for recognizing the response regulator and assisting transfer of the phosphoryl group. P3 corresponds to the linking domain, through which two SpaK subunits may form a dimer. P4 resembles the ATP binding domain, which autophosphorylates the conserved histidine residue. In histidine kinases most of the residues around the ATP binding site of the P4 unit are conserved, especially those comprising the characteristic sequence motifs (identified in Fig. 1B). In addition, the histidine kinase P4 unit has a loop-like lid (ATP lid) between the F and G2 boxes (corresponding to the SpaK model, residues 409 to 417), which controls the closed-to-open conformational change of the binding pocket. It is postulated that P5 acts as a regulative domain to modulate the activity of autotransphophorylation, responding to signals from the external environment [41].

To examine sequence homology in structure context between SpaK and various histidine kinases in the 5 “box” regions, we used LGA to globally align the SpaK homology model with all other histidine kinases from PDB that have these structure motifs. Structures with corresponding “box” regions included 2ftk_A, 1tid_A, 1b3q_A, and 2ch4_A. In Table 3 are shown structure-based alignments, including residue-residue correspondences, between our SpaK model (based on 2c2a) and 2ftk_A in the H-box regions, and between SpaK and 2ch4_A in the N-, G1-, F-, and G2-box regions. Calculated structural alignments between our SpaK model and the PDB structures (including those not shown) indicated significant structure conservation within these defined sequence motifs. The residue-residue correspondences arising from the LGA structure alignments were consistent with respect to highly conserved residues identified by Stock and coworkers [21] and by Grebe and Stock [43] (see bold-type residue-residue correspondences in Table 3), even in the more variable F-box regions. Within group HPK-3c, a small group of histidine kinases into which Grebe and Stock [43] classified SpaK, most histidine kinases have an F at the position corresponding to T404 in SpaK, whereas SpaK T404 corresponds to a T in some proteins in group HPK 1a. Furthermore, SpaK F407-Y408 has identity to the corresponding F-box FY in most proteins in group HPK 1a. As group HPK 3c is closely related to group HPK 1a, it is not surprising that there is ambiguity with respect to residue-residue correspondences within the relatively variable F box among the proteins in these two groups. Based on this ambiguity, we examined the alpha-carbon structure alignment between the SpaK model and 2ch4_A to verify that the side chains of the corresponding SpaK Y408 and 2ch4_A F491 were well aligned (not shown), which further supported the residue-residue correspondence between these two residues. Protein CheA (2ch4) is classified in group HPK 9, and as such the sequence alignment also shows an F in the position corresponding to SpaK Y408.

**Table 3:** Examples of pairwise residue-residue correspondences between SpaK, Beryllofluoride Spo0F, and CheA histidine kinase.

**“H box” motifs: 2ftk_A-SpaK (245–254)**					
**Res**	**ResName**	**Res**	**ResName**	**Distance**	**RMSD(3)**
S	28_A	L	245_A	0.411	0.076
R	29_A	A	246_A	0.532	0.071
**H**	30_A	**H**	247_A	0.597	0.149
D	31_A	E	248_A	0.668	0.119
W	32_A	I	249_A	0.949	0.064
M	33_A	K	250_A	1.52	0.329
N	34_A	I	251_A	1.505	0.044
K	35_A	P	252_A	1.523	0.207
L	36_A	I	253_A	1.299	0.106
Q	37_A	T	254_A	1.22	0.265
**“N box” motifs: 2ch4_A-SpaK (356–364)**					
**Res**	**ResName**	**Res**	**ResName**	**Distance**	**RMSD(3)**
L	403_A	L	356_A	0.48	0.172
L	404_A	L	357_A	0.67	0.163
H	405_A	N	358_A	0.716	0.183
L	406_A	I	359_A	0.512	0.159
L	407_A	L	360_A	0.334	0.271
R	408_A	T	361_A	0.564	0.289
**N**	409_A	**N**	362_A	0.558	0.277
A	410_A	A	363_A	0.623	0.202
I	411_A	V	364_A	0.615	0.33
**“G1 box” motifs: 2ch4_A-SpaK (387–396)**					
**Res**	**ResName**	**Res**	**ResName**	**Distance**	**RMSD(3)**
E	446_A	F	387_A	0.898	0.169
V	447_A	V	388_A	0.354	0.13
E	448_A	K	389_A	0.134	0.18
**D**	449_A	**D**	390_A	0.803	0.202
D	450_A	T	391_A	0.595	0.323
**G**	451_A	**G**	392_A	1.041	0.321
R	452_A	N	393_A	0.862	0.322
**G**	453_A	**G**	394_A	0.758	0.62
I	454_A	F	395_A	0.989	0.982
D	455_A	S	396_A	2.154	0.845
**“F box” motifs: 2ch4_A-SpaK (400–408)**					
**Res**	**ResName**	**Res**	**ResName**	**Distance**	**RMSD(3)**
L	483_A	L	400_A	0.819	2.499
N	484_A	K	401_A	1.193	0.703
F	485_A	K	402_A	1.008	0.233
L	486_A	A	403_A	0.84	0.306
**F**	487_A	T	404_A	0.987	0.45
V	488_A	E	405_A	1.894	0.474
P	489_A	L	406_A	2.433	0.365
G	490_A	**F**	407_A	2.514	0.611
**F**	491_A	Y	408_A	2.078	0.773
**“G2 box” motifs: 2ch4_A-SpaK (418–424)**					
**Res**	**ResName**	**Res**	**ResName**	**Distance**	**RMSD(3)**
S	501_A	G	418_A	3.312	1.066
G	502_A	H	419_A	0.966	1.007
R	503_A	Y	420_A	2.398	1.666
**G**	504_A	**G**	421_A	1.198	1.07
V	505_A	M	422_A	3.453	1.131
**G**	506_A	**G**	423_A	0.755	1.293
M	507_A	L	424_A	1.089	0.793

Comparisons are made in presumed functional “box” motifs, the highly conserved sequences termed H, N, G1, F, and G2 boxes, characteristic of histidine kinases [40]. 2ftk corresponds to Beryllofluoride (PDB: 2ftk) and 2ch4 corresponds to CheA histidine kinase (PDB: 2ch4). Highly conserved residues among the histidine kinase proteins are indicated in bold type [21],[43]. See Table 2 for column header abbreviations.

### In vitro phosphorylation of wild type SpaK and SpaR

**Figure 4:**
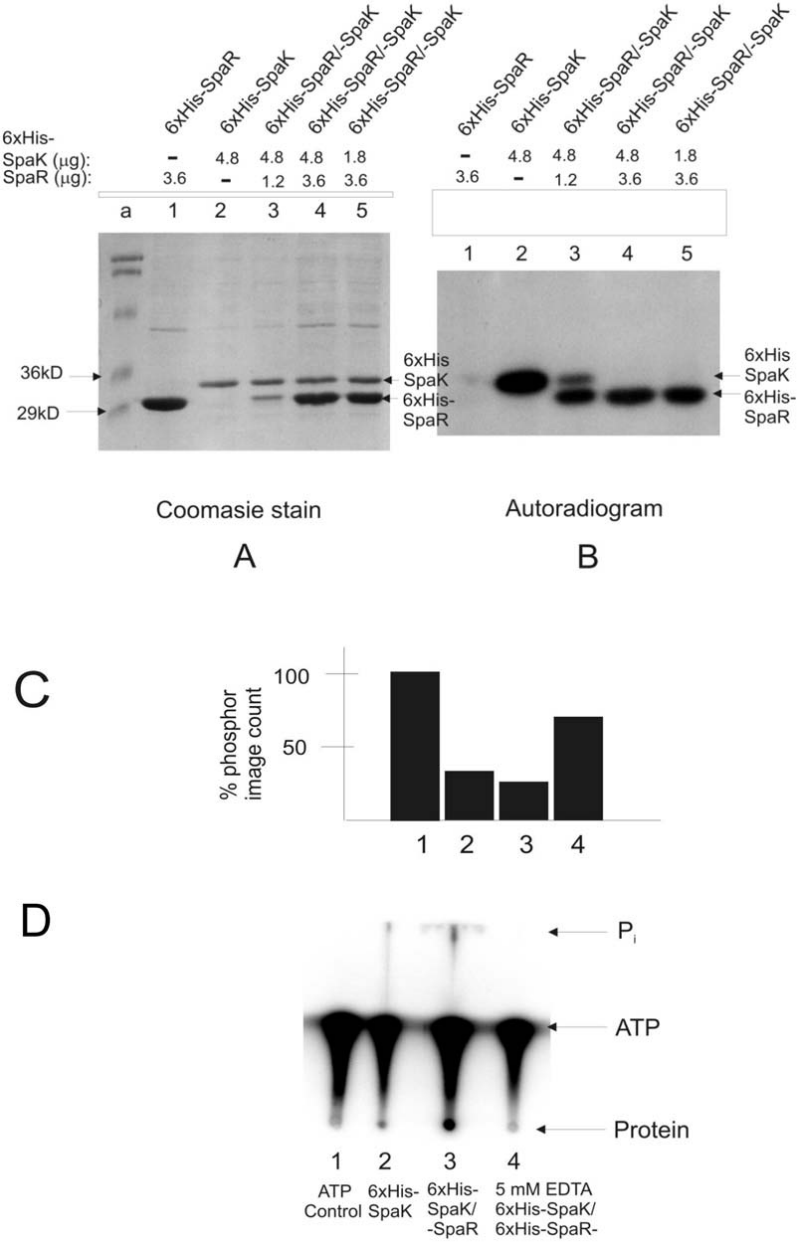
*In vitro* phosphorylation studies of SpaK and SpaR. A, B: SDS-PAGE of 6xHis-SpaK and 6xHis-SpaR in isolation or in combination and at various mass ratios, in the presence of ATP. A: Coomassie blue staining. B: Autoradiography; lane a: molecular weight markers. C: Phosphorimage analysis of SpaK incubated with [g-32P]-ATP (lane 1) followed by addition of 4 mM (lane 2), 10 mM (lane3), or 50 mM non-labeled (cold) ATP. D: PEI cellulose thin-layer chromatography of 6xHis-SpaK in isolation, or in combination with 6xHis-SpaR with and without EDTA.

To confirm whether SpaK undergoes auto-phosphorylation and subsequently transfers a phosphate moiety to SpaR, each protein was tested individually and in combination in the presence of radio-labeled ATP (Fig. 4). Combinations of 6xHis-SpaK and 6xHis-SpaR were created using 3 SpaK∶SpaR molar ratios of 4∶1, 4∶3, and 1∶2 shown in Fig. 4 A and B, lanes 3, 4, and 5, respectively. Only SpaK was phosphorylated in isolation (Fig. 4B lanes 1, 2), indicating that SpaK undergoes autophosphorylation. Phosphorylation of SpaR in the presence of SpaK (Fig. 4B lanes 3–5) indicated that phosphate is transferred from SpaK to SpaR. This transfer was incomplete at a molar ratio of SpaK∶SpaR of 4∶1, but reached completion at molar ratios of 4∶3 and 1∶2, indicating that transfer of phosphate from SpaK to SpaR reaches saturation as SpaK approaches molar equivalence or reaches molar excess relative to SpaR. These results imply that SpaR acts as a receptor for the phosphate group that is transferred from SpaK.

Quantification of radio-labeled phosphate-bound 6xHis-SpaK was performed to determine whether SpaK might exhibit phosphatase activity (Fig. 4C). Phosphor image analysis was used to measure the incorporation of radio-labeled phosphate by 6xHis-SpaK (Fig. 4C, histogram 1). This quantity served as baseline (100%) for comparison of 6xHis-SpaK samples that had been incubated in radio-labeled Pi followed by cold-ATP chase treatments (Fig. 4C, histograms 2–4). Cold chase with lower concentrations of ATP (4 mM or 10 mM) reduced the level of radio-labeled SpaK to levels about one-third to one-quarter that of the control, whereas a high concentration (50 mM) of unlabeled ATP resulted in a decrease in the rate of phosphate turnover, thereby reducing the level of radio-labeled SpaK only to about 70% that of the control. The decrease in the turnover of radio-labeled Pi on SpaK at high ATP concentration is suggestive of enzymatic inhibition of dephosphorylation (or phosphatase activity) rather than simple hydrolysis.

Thin-layer chromatography was performed to further examine the possibility that either SpaK or SpaR may exhibit phosphatase activity (Fig. 4D). Protein consisting of 6xHis-SpaK alone (Fig. 4D, lane 2) or 6xHis-SpaK in combination with 6xHis-SpaR (lane 3) was phosphorylated in the presence of radio-labeled ATP. In both cases, inorganic phosphate (Pi) was detected, but slightly more Pi and considerably more radio-labeled protein were detected when both proteins were present (compare Pi and Protein in lanes 2 and 3). The ATP-only control (lane 1) produced no detectable radio-labeled Pi, indicating that simple hydrolysis of ATP was not occurring. Furthermore, when phosphorylation was performed in the presence of EDTA, some phosphorylated protein was observed, although no inorganic phosphate was detected (Fig. 4D lane 4). This result, taken together with Fig. C, which suggested the presence of enzymatic phosphatase activity, supports the claim that SpaK (and possibly also SpaR) may possess enzymatic phosphatase activity.

### Mutational analysis of SpaK and intermolecular complementation of SpaK monomers

**Figure 5:**
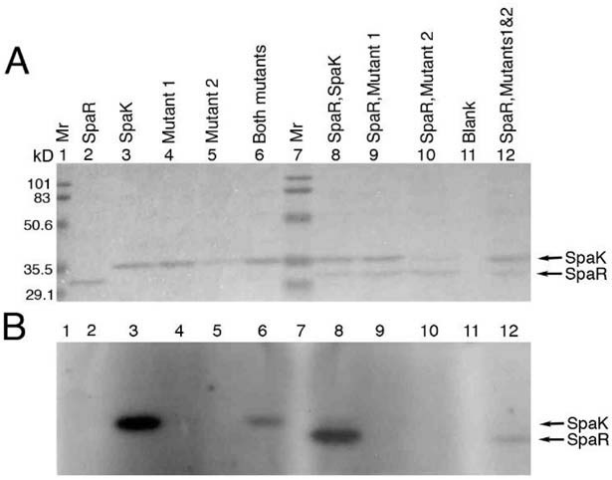
*In vitro* phosphorylation studies involving SpaK mutants. A, B: Polyacrylamide gel electrophoresis of 6xHis-SpaR and 6xHis-SpaK wild type or mutants in isolation or in combination, in the presence of ATP. Lanes 1, 7: molecular weight markers. A: Coomassie blue staining. B: Autoradiography. Mutant1: H247Q, Mutant 2: G392A.

Based on amino acid sequence alignment with other histidine kinases, the highly conserved histidine at position H247 was presumed to be the site of possible auto-phosphorylation, and a glycine located at position G392 in the C-terminal end of SpaK was determined to correspond to the conserved DXG motif of the nucleotide binding domain in related histidine kinases (Fig. 1A, Fig. 1B: H box and G1 box). In the superfamily of phosphotransferases, the conserved residues that form a corresponding motif (DXG in actin, GTG in hexokinase/glycerol kinase, and GNG in acetate and propionate kinases) are observed to be present in binding to a- and b-phosphate groups of the nucleotide [44]. Because several histidine kinases are believed to exist as homo-dimers and it is believed that phosphorylation occurs in trans, in which one monomer binds ATP in the nucleotide-binding domain and then transfers the phosphoryl group to a histidine located in the other monomer, we postulated that mutations at either of these positions might reduce or abolish auto-phosphorylation of SpaK, but that complementation between mutants might occur, effectively restoring function. We used site-directed mutagenesis to construct two mutants (see Materials and Methods): one in which the histidine at position H247 was changed to a glutamine (H247Q), and the other in which the glycine at position G392 was changed to alanine (G392A). Locations of mutated residues are shown in Fig. 1A. Phosphorylation studies of mutants H247Q and G392A revealed that both mutations resulted in loss of phosphorylation when each mutant was tested individually (Fig. 5 A, B; lanes 4, 5) or when individually combined with SpaR (Fig. 5B; lanes 9, 10). However, when the mutant proteins were combined, a detectable amount (approximately 25% that of wild type) of auto-phosphorylation was observed (Fig. 5B, lane 6), suggesting that complementation between the mutants had occurred, and supporting the hypothesis that SpaK forms a homo-dimer. Furthermore, when H247Q and G392A together were subjected to phosphorylation in the presence of wild type SpaR, the phosphoryl moiety was transferred to SpaR (Fig. 5B, lane 12).

## Discussion

In this work we demonstrated a quantitative approach for modeling protein-protein complexes using homology modeling followed by structure-based searches for multi-domain template proteins. In a search for templates upon which to base the model of a putative SpaK/SpaR complex, we used LGA, which applies two scoring schemes: GDT (global distance test) and LCS (longest continuous segment). Based on a previous study involving structure alignments between weakly homologous proteins [30], we applied a relatively stringent cutoff (LGA_S> = 35%)—Pettitt and coworkers [30] concluded that in order to assure the quality of a structure alignment between two domains, the GDT_TS score (a component of LGA's GDT) must exceed 25. In the current study we had observed a rapid increase in the number of hits obtained using LGA_S = 33% and below (not shown), and therefore we selected LGA_S = 35% as a conservative cutoff to assure confidence in selecting templates.

Although our approach can be used to identify domain-fusion protein structures that imply a possible functional association between two proteins of interest, it does not in itself provide sufficient information for modeling a physical interaction between the proteins. Protein domains that have less than 30–40% sequence homology to a “domain-fusion” template are likely to assume a similar orientation [8],[45]–but at sequence identity levels below this “interaction similarity twilight zone”, additional analysis is needed to make a reasonable prediction regarding the relative orientation of the interacting domains. In the current study, this additional analysis included identification and inspection of putative functional residues coupled with experimental analysis of mutant proteins. Thus, a protein-protein-complex model for a SpaK/SpaR interaction was initially built based on a structure-driven domain-fusion search method, followed by validation based on bioinformatic analysis and experimentation.

Our modeling effort supported the hypothesis that SpaK and SpaR may function as a histidine kinase sensor and a response regulator, respectively, in a two-component system. Based on homology modeling and domain-fusion analysis, residues corresponding to those believed to function in phosphorylation and subsequent transfer of a phosphate moiety from sensor to response regulator in other two-component systems were identified (Fig. 3, Tables 1, 2). Modeling of SpaK enabled structure comparisons with related sensor proteins (2ftk_A, 1tid_A, 1b3q_A, 2ch4_A), identification of sequences corresponding to the 5 highly conserved regions (“boxes”) that characterize class II two-component system proteins [40],[41],[43] (Table 3), and mapping of these sequences to the homology model of SpaK (Fig. 1B). Functional residues and conserved sequence motifs of our modeled SpaK/SpaR complex matched well with those of known sensor/response-regulator systems. Structure-based residue-residue correspondences (Tables 2, 3) agreed with sequence alignments used previously to classify histidine kinases [43],[46],[47], in which SpaK was placed in group HPK 3c in an 11-group classification by Grebe and Stock [43], but was unclassified according to the 5-type classification of Kim and Forst [46].

Phosphorylation studies of SpaK and SpaR showed that SpaK auto-phosphorylates and subsequently trans-phosphorylates SpaR (Fig. 4), confirming the hypothesis based on structure-driven domain-fusion analysis that SpaK and SpaR are functionally related and physically interact, and that the quaternary structure of the complex could enable transfer of a phosphate moiety between the protein subunits. Phosphorylation and complementation analyses using SpaK mutants suggested that residues H247 and G392 are important for auto- and trans-phosphorylation and that SpaK likely forms a dimer in which ATP binding and hydrolysis functions are split between the protomers (Fig. 5). Whereas both SpaK mutants (H247Q and G392A) were deficient in auto-phosphorylation (Fig. 5, lanes 4,5), this function was apparently restored when the mutants were combined (Fig. 5, lane 6), suggesting that complementation had occurred between the mutants. Complementation between H247Q and G392A also apparently restored trans-phosphorylation, as evidenced by phosphorylation of SpaR in the presence of both mutants (Fig. 5, lane 12). In an equimolar mixture of mutants H247Q and G392A, one would expect that approximately one-half of the resulting dimers would comprise a protomer of each mutant. Furthermore, phosphorylation would occur from the H247Q mutant to the G392A mutant, but not in the other direction, since G392A should not be able to bind ATP. Therefore the levels of auto-phosphorylation or trans-phosphorylation would not be expected to exceed one-half those of wild type SpaK. Also, although the H247Q/G392A mixed dimer may have had restored function, it would be expected to have functioned at less than the efficiency of a wild type SpaK dimer; since dimer formations between non-productive forms would occur, one would expect phosphorylation to proceed more slowly than in the wt. This is consistent with the observation that phosphorylation of or by H247Q combined with G392A (lanes 6, 12) occurred at levels considerably below those of wild type SpaK (lanes 3, 8).

In modeling the interaction between SpaK and SpaR we identified 6 suitable domain-fusion templates (Table 1), which were structurally clustered into two groups (see Results), each having a distinct conformation. Both groups displayed the same interaction pose with respect to the domain-domain interaction. Although each of the identified domain-fusion templates would have yielded a SpaK/SpaR complex model consistent with the experimental data, the criteria for selecting 2ftk as the domain-fusion template were based on combined structural identities between domains of 2ftk and the SpaK and SpaR models, on the resulting distance between putative functional residues involved in phosphate transfer (Fig. 3), and on the presence of a helical bundle domain, which enabled construction of a complete model. Interestingly, the domain-domain conformation between the helical bundle and the ATPase domains of 2c2a, used for modeling SpaK, differed from that of the corresponding domains within 2ftk. This difference suggests the possibility that a conformational change might take place when SpaK interacts with SpaR. Furthermore, it should be noted that the phospho-transfer in Spo0B-Spo0F (2ftk) occurs in the opposite direction (Asp to His) as that demonstrated here in SpaK-SpaR (Figs. 4, 5). This is not surprising, and does not diminish the value of 2ftk as a template for modeling a SpaK/SpaR interaction, given the considerable mechanistic diversity observed among structurally conserved domains comprising sensor/response-regulator systems [48].

Although structure modeling and experiments involving phophorylation studies strongly suggest functional and physical interactions between SpaK and SpaR, we cannot be entirely certain that our quaternary structure is correct with respect to domain composition, conformation, or orientation, as the methodology is dependent on existing structural data within PDB; it is possible that none of the domain-fusion templates detected by our approach is truly representative of the physical interaction between SpaK and SpaR, as homology modeling is, by definition, data driven. Due to the low sequence homologies between SpaK and SpaR and the identified domain-fusion templates, one could not conclude with any degree of certainty based solely on template identification that the interaction pose modeled here is likely to be correct [8]. However, combining bioinformatics analysis of known functional motifs (sequence “boxes”) and putative interacting residues with experimental evidence of function allows us to assert the value of the homology model of a putative SpaK/SpaR protein-protein complex. Our approach detects existing putative domain-fusion templates, which may suggest testable hypotheses regarding quaternary structure and function; a structure-based approach for identification of “Rosetta Stone” proteins greatly enhances structure-function hypothesis generation by providing structural context for putative functional residues. Additional bioinformatics analyses of a putative protein-protein complex model, which may verify the correctness of the model, include alignments of modified sequence profiles [7], for example, which use quantitative methods applied at the domain-domain interface to evaluate the likelihood of a stable interaction.

Although many two-component signal transduction systems have been identified by sequence homology, we wish to point out that a purely sequence-based approach would not have yielded the structural domain-fusion templates that were identified in this study. The strength of our approach is in its ability to identify putative domain-fusion templates based on structure homology searches in cases where sequence identities between the proteins of interest and the putative domain-fusion templates are low. Sequence identities of candidate domain-fusion templates to domains of SpaK and SpaR ranged from 4% to 25%, but in no instance was sequence identity greater than 7% simultaneously to both (Table 1). This point is emphasized by the lack of sufficient sequence-based evidence for linking these proteins using the standard domain-fusion approach: as of this writing, SpaK and SpaR are not linked in this way, for example, in Prolinks [5], nor did we find them linked by other sequence-based or empirical methods in DIP, BIND/BOND, MIPS, IntAct, MPIDB, or InterPreTS [49]–[54]. Homology modeling of SpaK and SpaR using a standard methodology [28] and subsequent structure-based searches using a quantitative structure comparison algorithm [20] is what enabled a more sensitive, structure-based homology search against PDB. In conclusion, our method provides a basis upon which a high-throughput system for identification of putative protein-protein interactions could be built on a whole-genome scale.

## Supporting Information

Figure S1 Construction of vectors for expression of SpaK and SpaR proteins. A) Expression vectors pQE-31-spaK. B) pQE-31-spaR.
(0.38 MB TIF)

Table S1 Candidate templates for homology modeling of SpaK monomer.
(0.06 MB PDF)

Table S2 Candidate templates for homology modeling of SpaR.
(0.06 MB PDF)

## References

[1] Kumar A, Snyder M (2002). Protein complexes take the bait.. Nature.

[2] Phizicky EM, Fields S (1995). Protein-protein interactions: methods for detection and analysis.. Microbiological Reviews.

[3] Shoemaker BA, Panchenko AR (2007). Deciphering protein-protein interactions. Part I. Experimental
Techniques and Databases.. PLoS Computational Biology.

[4] Uetz P, Glot L, Cagney G, Mansfield TA, Judson RS (2000). A comprehensive analysis of protein-protein interactions in
*Saccharomyces cerevisiae*.. Nature.

[5] Bowers PM, Pellegrini M, Thompson MJ, Fierro J, Yeates TO, Eisenberg D (2004). Prolinks: a database of functional linkages derived from
coevolution.. Genome Biology.

[6] Kundrotas PJ, Alexov E (2006). Predicting 3D structures of transient protein-protein complexes
by homology.. Biochimica et Biophysica Acta.

[7] Kundrotas PJ, Lensink MF, Alexov E (2008). Homology-based modeling of 3D structures of protein-protein
complexes using alignments of modified sequence profiles.. International Journal of Biological Macromolecules.

[8] Launay G, Simonson T (2008). Homology modeling of protein-protein complexes: a simple method
and its possibilities and limitations.. BMC Bioinformatics.

[9] Marcotte EM, Pelligrini M, Ng H-L, Rice DW, Yeates TO, Eisenberg D (1999). Detecting protein function and protein-protein interactions from
genome sequences.. Science.

[10] Pellegrini M, Marcotte EM, Thompton MJ, Eisenberg D, Yeates TO (1999). Assigning protein functions by comparative genome analysis:
protein phylogenetic profiles.. Proc Natl Acad Sci USA.

[11] Salwinski L, Eisenberg D (2003). Computational methods for protein-protein interaction analysis.. Current Opinion in Structural Biology.

[12] Szilaghyi A, Grimm V, Arakaki AK, Skolnick J (2005). Prediction of physical protein-protein interactions.. Physical Biology.

[13] Shoemaker BA, Panchenko AR (2007). Deciphering protein-protein interactions. Part II. Computational
methods to predict protein and domain interaction partners.. PLoS Computational Biology.

[14] Teichmann SA, Murzin AG, Chothia C (2001). Determination of protein function, evolution and interactions by
structural genomics.. Current Opinion in Structural Biology.

[15] Marcotte EM (2000). Computational genetics: finding protein function by nonhomology
methods.. Current Opinion in Structural Biology.

[16] Pace HC, Hodawadekar SC, Draganescu A, Huang J, Bieganovski P, Pekarsky Y, Croce CM, Brenner C (2000). Crystal structure of the worm NitFhit Rosetta Stone protein
reveals a Nit tetramer binding two Fhit dimmers.. Current Biology.

[17] Chia J-M, Kolatar PR (2004). Implications for domain fusion protein-protein interactions based
on structural information.. BMC Bioinformatics.

[18] Lu L, Lu H, Skolnick J (2002). MULTIPROSPECTOR: An algorithm for the prediction of
protein-protein interactions by multimeric threading.. Protein: Structure, Function, and Genetics.

[19] Rost B (1997). Protein structure sustain evolutionary drift.. Fold Des.

[20] Zemla A (2003). LGA—a method for finding 3D similarities in protein
structures.. Nucleic Acids Research.

[21] Stock J, Ninfa AJ, Stock AM (1989). Protein phosphorylation and regulation of adaptive response in
bacteria.. Microbiological Reviews, American Soc. Microbiol.

[22] Galperin MY (2006). Structural classification of bacterial response regulators:
Diversity of output domains and domain combinations.. Journal of Bacteriology.

[23] Kleerebezem M, Quadri LEN, Kuipers OP, de Vos WM (1997). Quorum sensing by peptide pheromones and two-component
signal-transduction systems in gram-positive bacteria.. Molecular Microbiology.

[24] Skerker JM, Prasol MS, Perchuk BS, Biondi EG, Laub MT (2005). Two-component signal transduction pathways regulating growth and
cell cycle progression in a bacterium: A system-level analysis.. PLoS Biology.

[25] Kleerebezem M, Bongers R, Rutten G, de Vos WM, Kuipers OP (2004). Autoregulation of subtilin biosynthesis in *Bacillus
subtilis*: the role of the spa-box in subtilin-responsive
promoters.. Peptides.

[26] Klein C, Kaletta C, Entian KD (1993). Biosynthesis of the lantibiotic subtilin is regulated by a
histidine kinase/response regulator system.. Applied and Environmental Microbiology.

[27] Stein T, Borchert S, Conrad B, Feesche J, Hofemeister B, Hofemeister J, Entian K-D (2002). Two different lantibiotic-like peptides originate from the ericin
gene cluster of *Bacillus subtilis* A1/3.. Journal of Bacteriology.

[28] Zemla A, Ecale Zhou C, Slezak T, Kuczmarski T, Rama D, Torres C, Sawicka D, Barsky D (2005). AS2TS system for protein structure modeling and analysis.. Nucleic Acids Research.

[29] Canutescu AA, Shelenkov AA, Dunbrack RL (2003). A graph theory algorithm for protein side-chain prediction.. Protein Science.

[30] Pettitt CS, McGuffin LJ, Jones DT (2005). Improving sequence-based fold recognition by use of 3D model
quality assessment.. Bioinformatics.

[31] Liu W, Hansen N (1991). Conversion of *Bacillus subtilis* 168 to a
subtilin producer by site-directed mutagenesis.. Journal of Bacteriology.

[32] Banerjee S, Hansen JN (1988). Structure and expression of a gene encoding the precursor of
subtilin, a small protein antibiotic.. Journal of Biological Chemistry.

[33] Buchman GW, Banerjee S, Hansen JN (1988). Structure, expression, and evolution of a gene encoding the
precursor of nisin, a small protein antibiotic.. Journal of Biological Chemistry.

[34] Satola S, Kirchman PA, Moran CP (1991). Spo0A binds to a promoter used by sigma^A^ RNA
polymerase during sporulation in *Bacillus subtilis*.. Proceedings of the National Academy of Science USA.

[35] Jiang M, Shao W, Perego M, Hoch JA (2000). Multiple histidine kinases regulate entry into stationary phase
and sporulation in *Bacillus subtilis*.. Molecular Microbiology.

[36] Zapf J, Sen MadhusudanU, Hoch J, Varughese K (2000). A transient interaction between two phosphorelay proteins trapped
in a crystal lattice reveals the mechanism of molecular recognition and
phosphotransfer in signal transduction.. Structure.

[37] Varughese KI, Tsigelny I, Zhao H (2006). The crystal structure of beryllofluoride Spo0F in complex with
the phosphotransferase Spo0B represents a phosphotransfer pretransition
state.. Journal of Bacteriology.

[38] Marina A, Waldburger C, Hendrickson WA (2005). Structure of the entire cytoplasmic portion of a sensor
histidine-kinase protein.. EMBO.

[39] Bilwes AM, Alex LA, Crane BR, Simon MI (1999). Structure of CheA, a signal-transducing histidine kinase.. Cell.

[40] Zhang W, Culley DE, Wu G, Brockman FJ (2006). Two-component signal transduction systems of
*Desulfovibrio vulgaris*: structural and phylogenetic
analysis and deduction of putative cognate pairs.. Journal of Molecular Evolution.

[41] Zhang J, Xu Y, Shen J, Luo X, Chen J, Chen K, Zhu W, Jiang H (2005). Dynamic mechanism for the autophosphorylation of CheH histidine
kinase: molecular dynamics simulations.. Journal of the American Chemical Society.

[42] Park SY, Borbat PP, Gonzalez-Bonet G, Bhatnagar J, Pollard AM, Freed JH, Bilwes AM, Crane BR (2006). Reconstruction of the chemotaxis receptor–kinase
assembly.. Nature.

[43] Grebe TW, Stock JF (1999). The histidine protein kinase superfamily.. Advances in Microbial Physiology.

[44] Simanshu DK, Savithri HS, Murthy MRN (2005). Crystal structures of ADP and AMPPNP-bound propionate kinase
(TdcD) from *Salmonella typhimurium*: comparison with members
of acetate and sugar kinase/heat shock cognate 70/actin superfamily.. Journal of Molecular Biology.

[45] Aloy P, Ceulemans H, Stark A, Russell RB (2003). The relationship between sequence and interaction divergence in
proteins.. Journal of Molecular Biology.

[46] Kim D-j, Forst S (2001). Genomic analysis of the histidine kinase family in bacteria and
archea.. Microbiology.

[47] Wolanin PM, Thomason PA, Stock JB (2002). Histidine protein kinases: key signal transducers outside the
animal kingdom.. Genome Biology.

[48] Gao R, Mack TR, Stock AM (2007). Bacterial response regulators: versatile regulatory strategies
from common domains.. TRENDS in Biochemical Sciences.

[49] Salwinski L, Miller CS, Smith AJ, Pettit FK, Bowie JU, Eisenberg D (2004). The database of interacting proteins: 2004 update.. Nucleic Acids Research.

[50] Alfarano C, Andrade CE, Anthony K, Bahroos N, Bajec M (2005). The biomolecular interaction network database and related tools
2005 update.. Nucleic Acids Research.

[51] Mewes HW, Frishman D, Mayer KF, Munsterkotter M, Noubibou O, Pagel P, Rattei T, Oesterheld M, Ruepp A, Stumpflen V (2006). MIPS: analysis and annotation of proteins from whole genomes in
2005.. Nucleic Acids Research.

[52] Kerrien S, Alam-Faruque Y, Aranda B, Bancarz I, Bridge A (2007). IntAct—open source resource for molecular interaction
data.. Nucleic Acids Research.

[53] Goll J, Rajagopala SV, Shiau SC, Wu H, Lamb BT, Uetz P (2008). MPIDB: the microbial protein interaction database.. Bioinformatics.

[54] Aloy P, Russel RB (2002). InterPreTS: protein interaction prediction through tertiary
structure.. Bioinformatics.

