# Supplementary figures and images for: SpaK/SpaR Two-component System Characterized by a Structure-driven Domain-fusion Method and in Vitro Phosphorylation Studies
Source: PLoS Comput Biol. 2009 Jun 5;5(6):e1000401. doi: 10.1371/journal.pcbi.1000401 (PMC2686270; doi:10.1371/journal.pcbi.1000401)

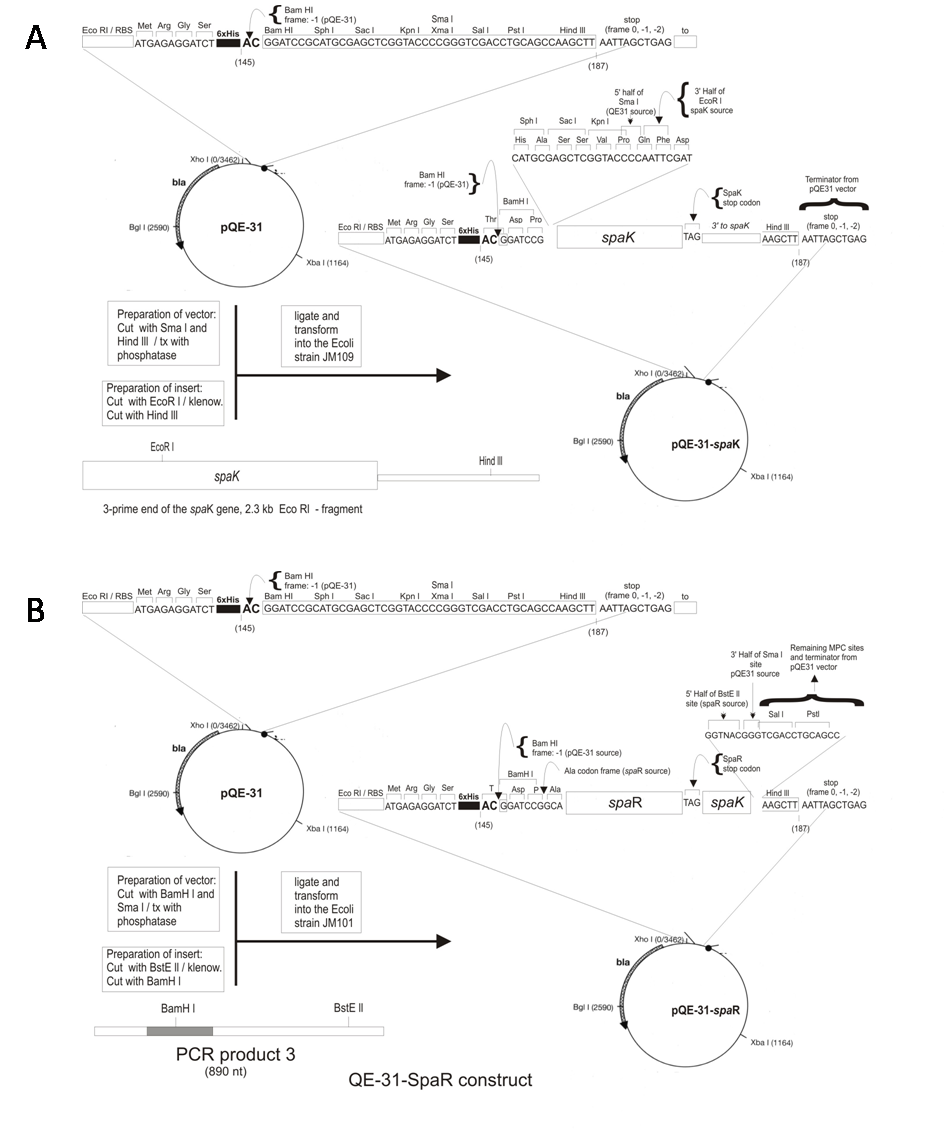

Supplement: Figure S1 — Construction of vectors for expression of SpaK and SpaR proteins. A) Expression vectors pQE-31-spaK. B) pQE-31-spaR. (0.38 MB TIF) [file pcbi.1000401.s001.tif]
