# Supplementary material for: SpaK/SpaR Two-component System Characterized by a Structure-driven Domain-fusion Method and in Vitro Phosphorylation Studies
Source: PLoS Comput Biol. 2009 Jun 5;5(6):e1000401. doi: 10.1371/journal.pcbi.1000401 (PMC2686270; doi:10.1371/journal.pcbi.1000401)
Supplement: Table S1 — Candidate templates for homology modeling of SpaK monomer. (0.06 MB PDF) [file pcbi.1000401.s002.pdf]

PDB	Length	E-value	Seq_ID	Coverage
2c2a_A	258	4.E-57	28	(229-455:11-246)
1r62_A	160	6.E-26	22	(311-458:9-160)
1bxd_A	161	1.E-22	21	(302-456:2-149)
1ysr_A	150	1.E-18	21	(306-455:4-147)
1id0_A	152	1.E-16	21	(304-452:2-140)
1i5d_A	191	4.E-10	17	(303-456:2-190)
1i58_A	189	9.E-10	17	(321-456:16-188)
2ch4_A	320	1.E-09	17	(321-456:16-188)
1l0o_A	150	4.E-07	16	(333-452:25-134)
1gkz_A	388	3.E-07	15	(310-454:195-370)
1b3q_B	379	2.E-07	14	(257-456:18-247)
1til_A	146	5.E-07	16	(333-452:25-134)
1th8_A	145	5.E-07	16	(333-452:25-134)
2btz_A	394	4.E-05	14	(310-448:182-341)
1tid_A	136	1.E-05	16	(334-452:26-134)
1y8o_A	419	4.E-04	13	(309-448:203-365)
1jm6_A	407	1.E-04	13	(310-448:195-354)
2e0a_A	394	3.E-22	13	(273-454:144-347)
3cgz_A	157	1.E-18	21	(305-454:4-147)
2q8g_A	407	2.E-08	15	(316-454:207-360)

Supplemental Table S1. Candidate templates for homology modeling of SpaK monomer. Coverage column indicates the ranges for which AS2TS identified acceptable residue-residue correspondences in the initially created alignment between SpaK sequence and the PDB template, respectively.
