# Supplementary material for: SpaK/SpaR Two-component System Characterized by a Structure-driven Domain-fusion Method and in Vitro Phosphorylation Studies
Source: PLoS Comput Biol. 2009 Jun 5;5(6):e1000401. doi: 10.1371/journal.pcbi.1000401 (PMC2686270; doi:10.1371/journal.pcbi.1000401)
Supplement: Table S2 — Candidate templates for homology modeling of SpaR. (0.06 MB PDF) [file pcbi.1000401.s003.pdf]

PDB	Length	E-value	Seq_ID	Coverage
1mvo_A	136	7.E-49	46	(2-126:4-130)
1p2f_A	220	5.E-52	30	(3-219:4-215)
2a9o_A	120	1.E-46	44	(1-119:1-120)
1ys7_B	233	1.E-61	28	(17-219:23-230)
2gwr_A	238	9.E-58	30	(3-218:7-224)
1kgs_A	225	3.E-61	33	(3-217:4-220)
2a9r_A	120	1.E-45	43	(1-119:1-120)
1b00_A	127	3.E-42	40	(1-120:1-125)
1zes_A	125	5.E-42	40	(1-120:1-125)
2iyn_B	124	1.E-41	40	(1-117:1-122)
1zh2_A	121	2.E-34	34	(1-120:1-121)
1xhe_B	123	1.E-32	29	(2-120:4-123)
1gxq_A	106	6.E-32	24	(119-219:1-102)
1xhf_B	123	3.E-32	29	(2-120:4-123)
2d1v_A	108	1.E-31	24	(120-217:1-100)
1zgz_A	122	1.E-31	31	(3-117:4-119)
1qqi_A	104	2.E-31	25	(124-219:4-100)
2oqr_A	230	4.E-79	29	(4-220:7-229)
2jba_A	127	8.E-40	39	(1-120:1-125)
2jb9_B	127	2.E-38	38	(1-120:1-125)

Supplemental Table S2. Candidate templates for homology modeling of SpaR. Coverage column indicates the ranges for which AS2TS identified acceptable correspondence between SpaR sequence and the PDB template, respectively.
